# Supramolecular Gel-Templated In Situ Synthesis and Assembly of CdS Quantum Dots Gels

**DOI:** 10.1186/s11671-016-1813-y

**Published:** 2017-01-13

**Authors:** Lili Zhu, Jie He, Xiaoliang Wang, Dawei Li, Haibing He, Lianbing Ren, Biwang Jiang, Yong Wang, Chao Teng, Gi Xue, Huchun Tao

**Affiliations:** 1 Guangdong Provincial Key Laboratory of Nano-Micro Material Research, School of Chemical Biology & Biotechnology, Peking University Shenzhen Graduate School, Shenzhen, 518055 China; 2 Department of Polymer Science and Engineering, The School of Chemistry and Chemical Engineering, Key Laboratory of High Performance Polymer Materials and Technology (Nanjing University), Ministry of Education, The State Key Laboratory of Coordination Chemistry, Nanjing University, Nanjing, 210093 China; 3 Key Laboratory for Heavy Metal Pollution Control and Reutilization, School of Environment and Energy, Peking University Shenzhen Graduate School, Shenzhen, 518055 China

**Keywords:** Quantum dots, Fluorescent probe, Self-assembled fibrillar networks, Template synthesis

## Abstract

Although many studies have attempted to develop strategies for spontaneously organizing nanoparticles (NPs) into three-dimensional (3D) geometries, it remains a fascinating challenge. In this study, a method for in situ synthesis and self-assembly of a CdS quantum dots (QDs) gel using a Cd supramolecular gel as a scaffold was demonstrated. During the QDs formation process, the Cd ions that constituted the Cd gels served as the precursors of the CdS QDs, and the oleic acid (OA) that ligated with the Cd in the supramolecular gels was capped on the surface of the CdS QDs in the form of carboxylate. The OA-stabilized CdS QDs were in situ synthesized in the entangled self-assembled fibrillar networks (SAFIN) of the Cd gels through reactions between the gelator and H_2_S. As a result, the QDs exactly replicated the framework of the SAFIN in the CdS QD gels instead of simply assembling along the SAFIN of the supramolecular gels. Moreover, the CdS QDs showed extraordinary sensitivity in the fluorescence detection of IO_4_^−^ anions. The facile one-step method developed here is a new approach to assembling nanostructured materials into 3D architectures and has general implications for the design of low molecular mass gelators to bring desired functionality to the developed supramolecular gels.

**Electronic supplementary material:**

The online version of this article (doi:10.1186/s11671-016-1813-y) contains supplementary material, which is available to authorized users.

## Background

Organized assemblies of nanoparticles (NPs) with diversified structures have received much attention because they combine both the unique size-dependent physical properties of individual NPs and the collective physical properties that can optimize and extend their applications in optical and electronic fields [1–10]. Various approaches have been developed to fabricate NPs into an ordered assembly, including self-assembly induced by cold treatment, solvent evaporation, and templated organization by biomolecules or lyotropic liquid crystals [11–29]. Despite these studies, developing a facile and efficient method to self-assemble NPs into ordered structures is still challenging, especially for three-dimensional (3D) geometries [30–35].

Recently, there has been considerable interest in using supramolecular gels, which are gels derived from low molecular mass gelators, to assemble NPs into different structures [36–45]. Due to the tunability of the structure of these gelators, the entangled self-assembled fibrillar networks (SAFINs) formed by the self-aggregation of these small gelators are superior templates for assembling NPs into two- or three-dimensional architectures [46–50]. Although a large number of studies of the in situ synthesis and stabilization of inorganic NPs are focused on Si/TiO_2_ NPs, an increasing number of studies have reported the ordered assembly of quantum dots (QDs) within a gel matrix [50–52]. Bardelang and Yu et al. reported the preparation of QD-dipeptide nanocomposite gels using an ultrasound technique [50]. The CdSe/ZnS QDs were found to form along the peptide fibers due to multiple weak van der Waals interactions. Simmons and John et al. introduced a method to incorporate nanoparticles into the strands of a gel by linking small molecules through non-covalent interactions [53]. The immobilization of the super-paramagnetic ferrite nanoparticles and photochromic CdS QDs confers magnetic and/or luminescent properties on the gels. Lu et al. demonstrated the self-organization of porous CdS nanofibers with “pearl-necklace” architectures using a dicholesterol-based organogelator as a template [51]. However, due to the difficulty in designing low-molecule mass gelators and the lack of a detailed understanding of the how these small gelators aggregate, it is still difficult to fabricate NPs into ordered architectures. In addition, in most previous studies NPs were attached to the fibers during the assembly process by interactions between the functional groups of protecting ligands on the NPs and the gelators. Few studies have reported the formation of NPs gels with 3D networks in which the NPs self-assembled to form the actual SAFIN of the NPs gels rather than only being coated on the fibers of the supramolecular gels.

In this study, a one-step method for the in situ synthesis of CdS QD gels with 3D networks using a supramolecular Cd organogel as a scaffold was developed. The morphology and the structure of the obtained CdS gels were investigated using transmission electron microscopy (TEM), X-ray diffraction (XRD), UV-visible, and Fourier transform infrared spectroscopy (FT-IR). Finally, we demonstrated that the CdS gels could serve as fluorescent probes for the selective determination of trace levels of IO_4_^−^ anions.

## Methods

### Chemicals and Materials

1-octadecene (ODE 90%), oleic acid (OA 90%), cadmium acetate (Cd(OAc)_2_.2H_2_O 99.99 + %), and sodium sulfide (>98%) were purchased from Aldrich Chemical Co. All of the solvents were commercially available and distilled before use. All of the glassware was cleaned and rinsed with Milli-Q water, and then dried in an oven overnight before use.

### Preparation of Cadmium Organogel

**Fig. 1:**
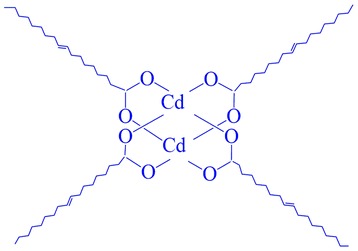
Structural formula of the organogel gelator

The organogel was prepared following Shi’s method [54]; 20 ml, 62.4 mmol ODE, 0.512 ml, 1.6 mmol OA, and 1.1064 g, 0.4 mmol cadmium acetate were mixed in a Schlenk flask. After degassing for 1 h at room temperature, the mixture was heated to 175 ^o^C with constant stirring in nitrogen. The reaction proceeded at 175 ^o^C for 1 h. Then the solution was left standing at room temperature to let the gelator molecules self-associate to form networks. The gelator was made of Cd complexes ligated with OLA (Cd_2_(OOC–(CH_2_)_7_–CH = CH–(CH_2_)_7_–CH_3_)_4_, as shown in Fig. 1.

### Preparation of CdS QDs Networks

The insitu synthesis of the three-dimensional networks was carried out by exposing the cadmium supramolecular organogel to H_2_S vapor, which was the source of the sulfur, at room temperature for 10 min. The H_2_S was generated from the neutralization of a saturated solution of sodium sulfide with 2.5 mol/L sulfuric acid. The reaction system was then left to stand at room temperature for 1 day. A saffron yellow CdS QD gel was obtained.

### Characterization

Purified samples of the CdS QDs were characterized using TEM, UV-vis absorption spectra, FT-IR, XRD, and fluorescence spectra. Acetone and chloroform were used to wash away any extra reactants. Then, the yellow CdS QDs were dispersed in toluene or dimethyl sulfoxide before further measurement.

All of the TEM images were recorded using a JEOL JEM-2100 electron microscopy at an accelerating bias voltage of 200 kV. The samples for the TEM analysis were prepared by dipping standard carbon-coated copper grids into the sample solution. The TEM grids were withdrawn from the solution and allowed to dry under ambient atmosphere overnight. FT-IR was performed with a Vector 22 FT-IR Spectrometer. The sample solution was deposited dropwise onto a KBr disc. The FT-IR spectra of the CdS QDs were collected after the evaporation of the solvents. The background spectra of a clean KBr disc were collected under the same experimental conditions and subtracted from the sample spectra. The UV-vis spectra were recorded in 200–600 nm range using a UV-3600 spectrophotometer, and the samples were dispersed in toluene with different concentrations. The fluorescence spectra were recorded with an Ls-55 fluorescence spectrometer from PE. The influence of the pH value and the concentration of CdS QDs on the fluorescence intensity were investigated. A phosphoric buffer solution (PBS) with a system pH value of around 7 was prepared by mixing Na_2_HPO_4_ · 2H_2_O and NaH_2_PO_4_ · 2H_2_O, and this solution was then used to tailor the PH of the sample solutions. The fluorescence intensity was recorded at 330 nm with an excitation wavelength of 273 nm. The XRD experiment was performed on a D8 ADVANCE X-ray powder diffractometer from Bruker AXS using CdS powder dried under a vacuum.

## Results and Discussion

**Fig. 2:**
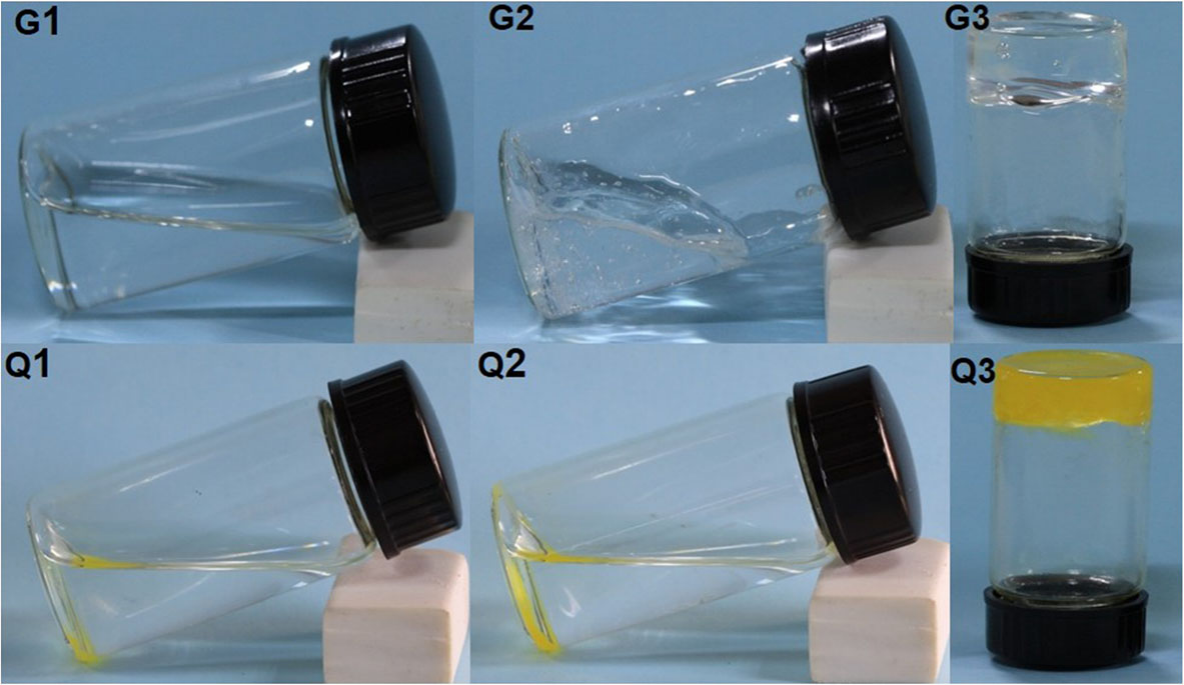
Photographs of Cd gelator samples: G1, G2, G3, and the corresponding CdS samples, Q1, Q2, and Q3

During the gelation process, the small gelator molecules self-aggregated to form SAFINs through a combination of non-covalent interactions. Figure 2 shows the samples gelated from the low-molecular mass gelator abbreviated as G1, G2, and G3, which were taken out after they had gelated for 8, 24, and 72 h, respectively. G1, G2, and G3, as they were taken out at different stages of the gelation process, were in different states. G1 and G2 were liquids, whereas G3 was an organogel. At the molecular level, the binuclear dicadmium tetraoleate (in Fig. 1) was assembled to form 3D networks in G3. Samples G1, G2, and G3 were exposed to H_2_S vapor to synthesize CdS QDs; the CdS samples obtained from G1, G2, G3 were abbreviated as Q1, Q2, and Q3, respectively. After reacting with H_2_S, all of the gelator samples changed from transparent to yellow or saffron yellow, indicating the formation of CdS QDs. As shown in Fig. 2, yellow precipitates were observed at the bottom in Q1 and Q2, because the CdS QDs precipitated from the ODE. However, in Q3 a saffron yellow gel formed rather than a precipitate, indicating that CdS QDs assembled into 3D networks in Q3. The Q3 samples remained stable at room temperature.

**Fig. 3:**
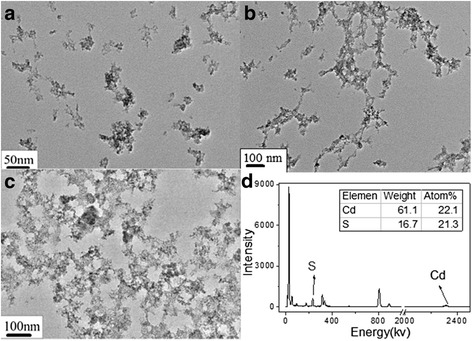
TEM images of CdS QDs from (**a**) Q1, (**b**) Q2, and (**c**) Q3. **d** EDS spectrum of CdS particles. Si and Pt signals were attributed to the material of the detecting instrument. CdS was placed on a piece of copper grid, which led to the observation of the Cu signal

The morphology of the CdS samples Q1, Q2, Q3 was studied by TEM, as shown in Fig. 3. Compared with the scattered structure of CdS in Q1 and Q2 (Fig. 3a, b), CdS gel sample Q3 had closely packed, networked structures (Fig. 3c). The TEM results demonstrated the existence of CdS QDs with network structures in Q3. Cadmium ions, which were constituents of the supramolecular gels, served as the precursors of CdS QDs. In addition, the formation of the gel in sample Q3 indicated the existence of interactions between OA-capped CdS QDs. Energy-dispersive X-ray spectroscopy (EDS) proved that Q3 was composed of Cd and S (Fig. 3d). Based on the EDS statistics, the molar ratio of Cd to S was 1:1.

**Fig. 4:**
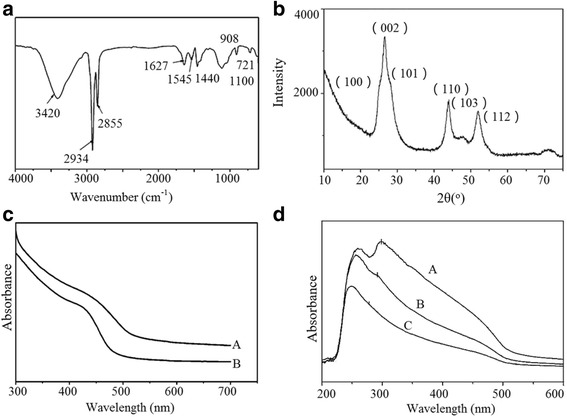
**a** FT-IR spectra of CdS QDs; **b** XRD patterns of vacuum-dried CdS powder; **c** UV-vis absorption spectra of CdS made from two samples in the process of gelation, gel(*B*) and gel(*A*); **d** UV-vis absorption spectra of Q3 with different concentrations of CdS QDs. A: 5.0 × 10^−3^ mol/l; B: 1.0 × 10^−3^ mol/l; C: 1.0 × 10^−4^ mol/l

FTIR was used to further investigate the interactions between OA and CdS QDs (shown in Fig. 4a). The absorptions at 2855 and 2934 cm^−1^ were assigned to the symmetric and asymmetric stretching vibrations of C–H, respectively. The disappearance of peaks for carboxylic at 1710 cm^−1^ and the appearance of peaks for carboxylate at 1627 and 1545 cm^−1^ indicated that OA reacted with CdS QDs and formed carboxylate. The peak at 721 cm^−1^ belonged to the rocking vibration of −CH_2_. These FTIR results confirmed that OA was successfully capped on the surface of CdS QDs. OA, which ligated with Cd in G3, capped on the surface of CdS QDs in the form of carboxylate. According to these results and the packing model of G3 reported above, we propose that OA-capped CdS QDs were in situ formed in the SAFINs of the supramolecular gel template, and they kept the exact morphology of the network structures. In a swollen state, the intermolecular interactions between such CdS building blocks led to a 3D network structure in Q3, which held sufficient ODE to form CdS QD gels.

The X-ray diffraction (XRD) results shown in Fig. 4b illustrated the refractions in the CdS hexagonal phase. The peaks located at 2θ = 26.5°, 44°, and 52° could be indexed to scattering from the (002), (110), and (112) planes, respectively. The peak at 26.5° was deconvoluted into three peaks viz. 24.9(100), 26.5(002), and 28.3(101). The fairly broad peaks confirmed the small particle size.

Macroscopically, apparent differences in color could be observed during the experiments. Compared with the CdS samples Q1 and Q2, the CdS gel Q3 was a darker color. As shown in Fig. 4c, UV-vis was adopted to investigate the assembly structure of Q2 (curve B) and Q3 (curve A). The UV-visible characterization of these composites revealed the presence of broad absorption bands with absorption maximums (*λ*_max_) at approximately 428 nm (curve A) and 418 nm (curve B). It manifested as a red shift in the UV-vis spectrum, caused by the formation of network structures. The absorption properties of CdS QDs from several Q3 samples at different concentrations were also investigated. As shown in the UV-vis spectra (Fig. 4d), along with an increase in CdS concentration in toluene, the absorption peak red-shifted from 273 to 301 nm. The peak at around 300 nm was a characteristic absorption of a magic-sized CdS nanocluster. The red shift should be attributed to the aggregation of CdS QDs. This result also proved the existence of the interactions between OA-capped CdS QDs.

**Fig. 5:**
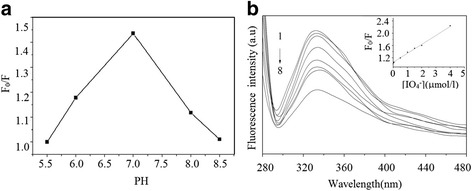
**a** Influence of pH on the relative fluorescence intensity. CdS QDs: 1.0 × 10^−5^mol/l. **b** The fluorescence spectra of CdS QDs in the presence of IO_4_^−^ at various concentrations (from 1 to 8): 0, 0.005, 0.05, 0.5, 1.0, 1.5, 2.0, and 4.0 μmol/l. *Inset*: calibration plots for IO_4_^−^

The use of QDs as selective fluorescent indicators for inorganic ions assay has been an active research field in analytical chemistry. However, most reports were concerned on the determination of metallic ions by using QDs as probes, few reports related to anion determination especially for the iodate anion. Iodine is an essential micronutrient in human growth. It is of great importance to develop efficient methods to detect iodate in salt and the environmental samples. Although many methods have been proposed, such as resonance scattering spectrometry and ion chromatography, it is still challenging to make the detection limits achieve at 10^−8^ mol/l. Here, it was demonstrated that OA-capped CdS QDs could be used as fluorescent probes to detect iodate anions (IO_4_^−^). The influence of the pH value on the fluorescence intensity was investigated (Fig. 5a). A phosphoric buffer solution (PBS) was used to keep the system pH value at around 7. The PBS was prepared by adjusting the amounts of Na_2_HPO_4_ · 2H_2_O and NaH_2_PO_4_ · 2H_2_O. The fluorescence intensity was recorded at 330 nm with an excitation wavelength of 273 nm. By varying the pH from 5.5 to 8.5, it was found that the maximum relative fluorescence intensity (*F*_0_/*F*) appeared at 7.0, where F_0_ was the fluorescence intensity of the QDs without quencher and F was the intensity with the quencher at a certain concentration. The decrease in acid medium might be caused by the protonation of QDs, whereas a high pH might result in the formation of cadmium hydroxide products. A PBS with pH 7 and 1.0 × 10^−5^ mol/l of CdS QDs solution (PBS with PH7) was chosen for further experiments.

As shown in Fig. 5b, the fluorescence intensity of CdS QDs was significantly quenched with increasing concentrations of IO_4_^−^ in the range from 0.005 to 4.0 μmol/l. The inset of Fig. 5b illustrates the observed good linear relationship between *F*_0_/*F* and the concentration of IO_4_^−^. The relationship can be described by the Stern-Volmer equation:

$$ {F}_0/F\kern0.5em =\kern0.5em 1+\mathrm{K}\mathrm{s}\mathrm{v}\left[\mathrm{Q}\right], $$

where Ksv is the Stern-Volmer quenching constant and [Q] is the concentration of the quencher. The experimental data obtained from IO_4_^−^ fitted the following equations:

$$ {\mathrm{IO}}_{4^{{}^{-}}}:\kern0.5em {F}_0/F\kern0.5em =\kern0.5em 1.0454+2.99\times {10}^5\left[{\mathrm{IO}}_{4^{{}^{-}}}\right], $$

with a correlation coefficient (R) of 0.99583.

The limit of detection (LOD) is defined by the equation LOD = 3S_0_/K, where *S*_0_ is the standard deviation of blank measurements and *K* is the slope of the calibration graph. For IO_4_^−^, the LOD was 3.5 × 10^−8^ mol/l.

Recent studies have tried to find a facile method for constructing NPs into an ordered assembly, especially a method that can stably incorporate the NPs in the networks and form NPs gels. In this study, OA ligated with Cd ions and formed a low-molecule mass gelator of Cd supramolecular gels. By simply introducing sulfur, OA-capped CdS was synthesized in situ. The stabilized layer of OA on the surface of the QDs and the core CdS exactly mirrored the gelators. The in situ formation and the small size of the QDs ensured that the CdS QDs not only retained the 3D network structures of the Cd gels, but also that they existed in gel form. In the obtained CdS gels, the CdS QDs were not assembled along the fiber of the networks, but rather exactly constituted the frameworks. Notably, the formation of the CdS QD gels was greatly influenced by the experimental and environmental parameters (see Additional file 1: Figure S1), such as the air humidity and H_2_S gas velocity. The new approach developed here points to a new direction for designing proper gelators for further applications. Moreover, we demonstrated that the obtained CdS QDs could be used as fluorescence probes for IO_4_^−^. This expands on the applications of previous QDs, which were used in the determination of metallic ions and, rarely, anion determination.

## Conclusion

In summary, a facile one-step method for the in situ synthesis of CdS QD gels at room temperature is presented, using a Cd supramolecular gel as a scaffold. Cd ions from the origin supramolecular gels were used as the precursors of CdS QDs. It was demonstrated that CdS QDs replicated and retained the 3D network structures of the supramolecular gels during the self-assembly process. OA capped on the surface of CdS QDs, not by physical adsorption, but in the form of carboxlate. The resulting QDs can be used as highly sensitive fluorescence probes to detect anions. The present approach offers a new simple method for fabricating QDs with 3D networks, which is a potential route to constructing electronic and optical devices.

## Additional file

Additional file 1: Figure S1. FT-IR spectra of native gel (*red line*) and dehydrated solution sample after put in drier for 1 month (*black line*). (DOCX 70 kb)

## Abbreviations

3D: Three-dimensional
FT-IR: Fourier transform infrared spectroscopy
NPs: Nanoparticles
PBS: Phosphoric buffer solution
QDs: Quantum dots
SAFIN: Self-assembled fibrillar networks
TEM: Transmission electron microscopy
XRD: X-ray diffraction
