# Supplementary material for: Supramolecular Gel-Templated In Situ Synthesis and Assembly of CdS Quantum Dots Gels
Source: Nanoscale Res Lett. 2017 Jan 13;12:30. doi: 10.1186/s11671-016-1813-y (PMC5236034; doi:10.1186/s11671-016-1813-y)
Supplement: Additional file 1: Figure S1. — FT-IR spectra of native gel (red line) and dehydrated solution sample after put in drier for 1 month (black line). (DOCX 70 kb) [file 11671_2016_1813_MOESM1_ESM.docx]

**Supramolecular gel-templated in-situ synthesis and assembly of CdS quantum dots gels**

Lili Zhu,^a,c^ Jie He,^a^ Xiaoliang Wang,^b^ Dawei Li,^b^ Haibin He,^b^ Lianbing Ren ,^a^ Biwang Jiang,^a^ Yong Wang,^a*^ Chao Teng,^a*^ and Gi Xue^b＊^and Huchun Tao,^c＊^

1. Guangdong Provincial Key Laboratory of Nano-Micro Material Research, School of Chemical Biology & Biotechnology, Peking University Shenzhen Graduate School, Shenzhen 518055, China.
2. Department of Polymer Science and Engineering, The School of Chemistry and Chemical Engineering, Key Laboratory of High Performance Polymer Materials and Technology (Nanjing University), Ministry of Education, The State Key Laboratory of Coordination Chemistry, Nanjing University, Nanjing 210093, China.
3. Key Laboratory for Heavy Metal Pollution Control and Reutilization, School of Environment and Energy, Peking University Shenzhen Graduate School, Shenzhen 518055, China.

To investigate the effect of trace amount of water on the formation of supramolecular gel, we performed the dehydrated experiments. The cadmium acetate was pretreated, heated at 165 ºC for about three hours until the powder turned light yellow, to remove trace water. The solution product was put in a drier after synthesis. It showed no indication of gelation, just behaving as liquid. Then we added [micro](http://www.iciba.com/micro/) amount of [water](http://www.iciba.com/water/) to it, significant [trend](http://www.iciba.com/trend/)ency to form gel could be observed just several hours later. It was obvious that a minute quantity of water tremendously accelerated the gelation of the supramolecular gel and played a critical role during gelation. We conceived that hydrogen bond provided by water was the key factor in forming three-dimensional networks.

Moreover, we performed the FT-IR experiment to compare the native gel and the hydrated samples (shown in Figure S1). In native gel, peaks at 3400 and 3610 cm^-1^ are assigned respectively to hydrogen bond and dissociative O-H bond. Peaks at 1533 cm^-1^ are assigned to the carboxylate stretching bands. A noticeable difference in dehydrated samples was that the carboxylate stretching bands shifted to 1542 cm^-1^, which could be assigned to the effect of hydrogen bond between water and C=O. At the same time, peaks at 3400 and 3610 cm^-1^ disappeared. According to the FT-IR results, it is ascertainable hydrogen bond provided by the micro amount of water interacts with carboxylate groups and promotes the gelation.

Figure S1. FT-IR spectra of native gel (red line) and dehydrated solution sample after put in drier for one month (black line).
